# Cardiac-targeted *PIASy* gene silencing mediates deSUMOylation of caveolin-3 and prevents ischemia/reperfusion-induced Na_v_1.5 downregulation and ventricular arrhythmias

**DOI:** 10.1186/s40779-022-00415-x

**Published:** 2022-10-14

**Authors:** Chen-Chen Hu, Xin Wei, Jin-Min Liu, Lin-Lin Han, Cheng-Kun Xia, Jing Wu, Tao You, A.-Fang Zhu, Shang-Long Yao, Shi-Ying Yuan, Hao-Dong Xu, Zheng-Yuan Xia, Ting-Ting Wang, Wei-Ke Mao

**Affiliations:** 1 Department of Anesthesiology, Institute of Anesthesiology and Critical Care Medicine, Union Hospital, Tongji Medical College, Huazhong University of Science and Technology, Wuhan, 430022 China; 2 Department of Cardiology, the Second Affiliated Hospital of Soochow University, Suzhou, 215004 Jiangsu China; 3 Department of Anesthesiology, Peking Union Medical College Hospital, CAMS and PUMC, Beijing, 100730 China; 4 Department of Pathology, University of Washington, Seattle, WA 98195 USA; 5 State Key Laboratory of Pharmaceutical Biotechnology, the University of Hong Kong, Hong Kong, 999077 China; 6 Department of Anesthesiology, Affiliated Hospital of Guangdong Medical University, Zhanjiang, 524000 Guangdong China

**Keywords:** Ventricular arrhythmia, Na_v_1.5, Caveolin-3, Protein inhibitor of activated STAT Y, SUMOylation

## Abstract

**Background:**

Abnormal myocardial Na_v_1.5 expression and function cause lethal ventricular arrhythmias during myocardial ischemia–reperfusion (I/R). Protein inhibitor of activated STAT Y (PIASy)-mediated caveolin-3 (Cav-3) SUMO modification affects Cav-3 binding to the voltage-gated sodium channel 1.5 (Na_v_1.5). PIASy activity is increased after myocardial I/R, but it is unclear whether this is attributable to plasma membrane Na_v_1.5 downregulation and ventricular arrhythmias.

**Methods:**

Using recombinant adeno-associated virus subtype 9 (AAV9), rat cardiac *PIASy* was silenced using intraventricular injection of *PIASy* short hairpin RNA (shRNA). After two weeks, rat hearts were subjected to I/R and electrocardiography was performed to assess malignant arrhythmias. Tissues from peri-infarct areas of the left ventricle were collected for molecular biological measurements.

**Results:**

PIASy was upregulated by I/R (*P* < 0.01), with increased SUMO2/3 modification of Cav-3 and reduced membrane Na_v_1.5 density (*P* < 0.01). AAV9-*PIASy* shRNA intraventricular injection into the rat heart downregulated PIASy after I/R, at both mRNA and protein levels (*P* < 0.05 vs. Scramble-shRNA + I/R group), decreased SUMO-modified Cav-3 levels, enhanced Cav-3 binding to Na_v_1.5, and prevented I/R-induced decrease of Na_v_1.5 and Cav-3 co-localization in the intercalated disc and lateral membrane. *PIASy* silencing in rat hearts reduced I/R-induced fatal arrhythmias, which was reflected by a modest decrease in the duration of ventricular fibrillation (VF; *P* < 0.05 vs. Scramble-shRNA + I/R group) and a significantly reduced arrhythmia score (*P* < 0.01 vs. Scramble-shRNA + I/R group). The anti-arrhythmic effects of *PIASy* silencing were also evidenced by decreased episodes of ventricular tachycardia (VT), sustained VT and VF, especially at the time 5–10 min after ischemia (*P* < 0.05 vs. Scramble-shRNA + IR group). Using in vitro human embryonic kidney 293 T (HEK293T) cells and isolated adult rat cardiomyocyte models exposed to hypoxia/reoxygenation (H/R), we confirmed that increased PIASy promoted Cav-3 modification by SUMO2/3 and Na_v_1.5/Cav-3 dissociation after H/R. Mutation of SUMO consensus lysine sites in Cav-3 (K38R or K144R) altered the membrane expression levels of Na_v_1.5 and Cav-3 before and after H/R in HEK293T cells.

**Conclusions:**

I/R-induced cardiac PIASy activation increased Cav-3 SUMOylation by SUMO2/3 and dysregulated Na_v_1.5-related ventricular arrhythmias. Cardiac-targeted *PIASy* silencing mediated Cav-3 deSUMOylation and partially prevented I/R-induced Na_v_1.5 downregulation in the plasma membrane of cardiomyocytes, and subsequent ventricular arrhythmias in rats. PIASy was identified as a potential therapeutic target for life-threatening arrhythmias in patients with ischemic heart diseases.

**Supplementary Information:**

The online version contains supplementary material available at 10.1186/s40779-022-00415-x.

## Background

Cardiac dysrhythmias are common causes of morbidity and mortality in patients with heart diseases. Ion channel alterations play pivotal roles in the development of cardiac arrhythmias. Voltage-gated sodium channel 1.5 (Na_v_1.5) is encoded by *SCN5A*; it is an α-subunit of a cardiac Na^+^ channel [1] and determines heart excitability and conduction [2]. Dysregulated Na^+^ channel expression was reported in several cardiac pathophysiological conditions such as myocardial infarction, heart failure, and other heart diseases, leading to life-threatening arrhythmias [3]. Dysfunctional Na^+^ channels, resulting from *SCN5A* mutations, are linked to Brugada syndrome, long QT syndrome, cardiac conduction defects, and atrial fibrillation [4]. Under normal circumstances, Na^+^ channel activity is finely regulated by complex molecular mechanisms. Accumulating evidence now indicates that multiple proteins, including ankyrin-G [5], caveolin-3 (Cav-3), E3 ubiquitin ligase NEDD4 [6], MOG1 [7], syntrophin [8], and dystrophin [9] interact with Na_v_1.5 and contribute to its function [10].

One such Na_v_1.5 interacting protein, Cav-3, is encoded by *CAV3,* appears to regulate Na_v_1.5 expression and function, and is a major component of caveolin subtypes including Cav-1α, -1β, -2α, -2β, -2γ, and -3 [11]. Recent findings indicated that several cardiac ion channels, such as Na_v_1.5, may be integrated into specific macromolecular signaling complexes for improved regulation [12]. Cav-3 is predominantly expressed in skeletal and cardiac myocytes and regulates Na_v_1.5 in caveolae by inhibiting neuronal nitric oxide synthase-dependent Na_v_1.5 S-nitrosylation [13]. Cav-3 mutations have been implicated in type 9 long QT syndrome and sudden infant death syndrome, with increased late sodium current [14]. However, the molecular mechanisms whereby Cav-3 interacts and regulates Na_v_1.5 activity remain largely unknown.

SUMOylation is a reversible post-transcriptional protein modification by small ubiquitin-like modifier (SUMO) proteins, which dynamically mediate functional changes in SUMO-targeted proteins [15]. SUMOylation biochemical processes are mediated by E1, E2, and E3 enzymes, leading to altered protein–protein interactions, subcellular localization, or target protein degradation [16]. Previously it was reported that Cav-3 SUMOylation putatively affected β-adrenergic receptor expression and desensitization [17].

The mammalian protein inhibitor of activated STAT (PIAS) protein represents a SUMO E3 ligase composed of four members: PIAS1, PIAS2, PIAS3, and PIASy [18]. As an important SUMO E3 ligase, PIASy helps regulate several metabolism-related proteins, such as peroxisome proliferator-activated receptor, adenosine 5’-monophosphate-activated protein kinase, and sirtuin [19, 20]. Cav-3 is also targeted by and interacts with PIASy, in heterogeneous transfected cells and cardiomyocytes [17]. In our previous research, we identified decreased membrane Na_v_1.5 density upon cardiac ischemia/reperfusion (I/R), which was related to cardiac arrhythmias [21]. Although dysfunctional Na_v_1.5 under I/R caused lethal cardiac arrhythmias, it was unclear if activated Cav-3 SUMOylation by PIASy alters Na_v_1.5 abundance after I/R.

In this study, adeno-associated virus subtype 9 (AAV9)-transferred *PIASy* short hairpin RNAs (shRNAs) were used in in vivo myocardial I/R rat and in vitro hypoxia/reoxygenation (H/R) models in isolated adult rat cardiomyocytes and human embryonic kidney 293 T (HEK293T) cells transfected with SUMO machinery, to examine whether enhanced PIASy activity-mediated Cav-3 SUMOylation after myocardial I/R contributes to plasma membrane Na_v_1.5 downregulation and ventricular arrhythmias.

## Methods

### Animals and cell lines

Animal studies were performed in strict accordance with Care and Use of Laboratory Animal guidelines formulated by the National Institutes of Health (NIH, Bethesda, MD, USA). Protocols were approved by the Animal Care and Use Committee of Tongji Medical College of Huazhong University of Science and Technology ([2022] IACUC Number: 2853).

We used 8–10 weeks old male Sprague Dawley rats, weighing 200–300 g (Wuhan University Laboratory Animal Center, Wuhan, China). The HEK293 cell line (American Type Culture Collection, Rockville, MD) was cultured in Dulbecco’s Modified Eagle’s Medium (Gibco, Grand Island, NY, USA) supplemented with 10% fetal bovine serum (Gibco, Grand Island, NY, USA) and 100 mg/ml sodium pyruvate (Gibco, Grand Island, NY, USA) in a humidified atmosphere containing 5% CO_2_ and 95% air at 37 ℃. Adult rat cardiomyocytes were isolated using the Langendorff method and cultured as previously described [22]. Rat hearts were rapidly removed after anaesthetization and hanged in the Langendorff apparatus. Hearts were then retroperfused and digested with collagenase II (0.8 mg/ml; Sigma-Aldrich, USA) and protease (0.1 mg/ml; Sigma-Aldrich, USA). Cardiomyocytes were purified using natural sedimentation techniques and cultured in serum-free DMEM in laminin-covered (Sigma-Aldrich, USA) dishes.

### Construction and in vivo cardiac transfection of AAV9-ZsGreen-shRNA

AAV9-ZsGreen vectors carrying *PIASy*-shRNA or scramble-shRNA were synthesized by Shenzheng BioWit Technologies Co., Ltd., (Shenzhen, China) with titers of 1.0 × 10^12 ^vg/ml. Briefly, three shRNAs targeting *PIASy* were designed and three plasmids were constructed. After enzyme digestion and sequencing, plasmids were packaged to AAV9 harboring ZsGreen and viruses were harvested. Based on quantitative-polymerase chain reaction data, the shRNA plasmid with the best interfering efficiency was selected, and virus purification and titer determination were performed following standard protocols.

Cardiac AAV9 vector delivery in vivo was performed by intraventricular injection into 8-week-old male Sprague–Dawley rats according to a previous protocol [23]. Rats were anesthetized with intraperitoneal pentobarbital sodium (70 mg/kg) and fixed in a supine position. An insulin syringe was inserted through the thoracic wall into the left ventricular cavity and positioning was confirmed by blood withdrawal. Then, an AVV9 suspension (50 μl, 1.3 × 10^11^ vg/heart) was slowly injected.

### Animal groups

We randomly assigned 80 rats to four groups (*n* = 20 per group): (1) Scramble-shRNA (AAV9 ZsGreen scramble shRNA transfer with sham operation), (2) Scramble-shRNA + I/R (AAV9 ZsGreen scramble shRNA transfer with I/R), (3) *PIASy*-shRNA (AAV9 ZsGreen *PIASy* shRNA transfer with sham operation), and (4) *PIASy*-shRNA + I/R (AAV9 ZsGreen *PIASy* shRNA transfer with I/R). Rats were subjected to I/R 14 d after AAV9 shRNA intraventricular injection.

### In vivo I/R and in vitro H/R models

An in vivo myocardial I/R model was established by surgical ligation of the left anterior descending coronary artery (LAD) as previously described [21]. Rats in Scramble-shRNA and *PIASy*-shRNA groups underwent the same procedure except for LAD ligation. Two weeks after systemic AAV9 vector delivery, animals were subjected to 45 min ischemia followed by 2 h reperfusion. Fresh ventricular tissue samples in peri-infarct regions (an approximately 3 mm area surrounding the infarction induced by LAD ligation) were obtained, snap frozen in liquid nitrogen, and stored at − 80 °C.

An in vitro simulated I/R model was established by exposing transfected HEK293 cells to hypoxia (serum-free DMEM in a humidified atmosphere + 1% air + 5% CO_2_ + 94% N_2_) for 3 h followed by 2 h reoxygenation (DMEM with serum in 5% CO_2_ + 95% air). Also, isolated adult rat cardiomyocytes were exposed to hypoxia for 40 min followed by 30 min reoxygenation.

### Plasmids and transfections

The pTracer-SV40 plasmid containing wild-type (WT) human *Na*_*v*_*1.5* was kindly provided by Dr. Thomas Zimmer at Friedrich Schiller University Jena, Thuringia, Germany. Flag-tagged Cav-3 (human Cav-3) was purchased from Origene Technologies (Rockville, USA). Flag-hPIASy, HA-SUMO1, HA-SUMO2/3, ubiquitin-conjugating enzyme 9 (ubc9), and scramble pCMV6-entry plasmids were obtained from Addgene (Watertown, MA, USA). Cav-3 mutations at K38 and K144 positions (Flag-tagged) were produced and verified by Shanghai Genechem Co., Ltd. (Shanghai, China). Plasmids, including the SUMO machinery (SUMO2/3, SUMO1, ubc9, and PIASy), Cav-3 (WT, K38R, and K144R mutants), and Na_v_1.5, were transfected into cells using Attractene from Qiagen (Hilden, Germany), following manufacturer’s instructions. HEK293 cells transfected with different plasmids were exposed to hypoxia or H/R to assess Cav-3 binding to SUMO2/3 and Na_v_1.5, respectively.

### Immunofluorescence and histology

Two weeks after AAV9 vector transfer of the reporter gene (ZsGreen) into rat myocardium, 6 μm cryosections were prepared from rat heart, lung and liver tissues, and examined for green fluorescence protein expression under fluorescence microscopy (Olympus BX-51 microscope, Olympus, Melville, NY, USA). Transfected HEK293 cells, isolated adult rat cardiomyocytes exposed to H/R, and fresh frozen ventricular sections from study groups were fixed for 10 min in 4% formaldehyde (Beyotime Biotechnology, Shanghai, China), stained with primary antibodies targeting PIASy (sc-166706, sc-50348, Santa Cruz Biotechnology, Danvers, MA, USA) and Cav-3 (ab2912, Abcam, Cambridge, MA, USA), Na_v_1.5 (#ASC-005, Alomone Lab, Jerusalem, Israel), and SUMO2/3 (ab3754, Abcam, Cambridge, MA, USA), followed by incubation with goat anti-mouse IgG secondary antibody (#4408, Santa Cruz Biotechnology, Danvers, MA, USA) or goat anti-rabbit IgG secondary antibody (#8889, Cell Signaling Technology, Danvers, MA, USA). Nuclei were stained with 4′6-diamidino-2-phenylindole (DAPI, C1005, Beyotime Biotechnology) during secondary antibody incubations. Fresh ventricular tissues were paraffin-embedded, and 5 μm sections were stained with hematoxylin and eosin (H&E). An Olympus fluorescence microscope was used for imaging.

### RNA extraction and quantitative real-time PCR (qRT-PCR)

Total RNA was extracted from ventricular tissue samples using TRIzol reagent (15596026, Invitrogen, Carlsbad, CA) and treated with DNase I to remove genomic DNA. We performed qRT-PCR on a Bio-Rad thermocycler (Bio-Rad, Hercules, USA) using a SYBR green kit (Invitrogen, Carlsbad, CA) following manufacturer’s instructions. Amplification primers were: Na_v_1.5, forward 5’-CCTTCACTGCCATCTACAC-3’ and reverse 5’-GCCTGAAATGACCGATAT-3’; Cav-3, forward 5’-GACATTGTGAAGGTGGATTT-3’, and reverse 5’-GTAGACAGCAGGCGGTAG-3’; GAPDH, forward 5’-AAGGGCTCATGACCACAGTC-3’ and reverse 5’-GGATGCAGGGATGATGTTCT-3’ (Wuhan BioBuffer Biotechnology, Co. Ltd., Wuhan, China).

### Co-immunoprecipitation (Co-IP) and western blotting

The following primary antibodies were used for Western blotting and Co-IP: anti-SUMO1 (ab11672, 1:500), anti-SUMO2/3 (ab3754, 1:500), anti-Cav-3 (rabbit polyclonal, ab2912, 1:1000), and anti-sodium potassium ATPase (Na^+^/K^+^ ATPase) antibody (ab198366, 1:1000) from Abcam (Cambridge, MA, USA). Anti-phospho-tyrosine (#9411, 1:500), anti-phospho-threonine antibody (#9381, 1:500), anti-GFP (#2555, 1:1000), and anti-β-actin (#4970, 1:2000) from Cell Signaling Technology (Danvers, MA, USA). Anti-Cav-3 (mouse polyclonal, sc-55518, diluted 1:1000) and anti-PIASy (sc-166706, sc-50348, 1:1000) from Santa Cruz Biotechnology (Dallas, Texas, USA). Anti-Na_v_1.5 (#ASC-005, 1:250) from Alomone Labs (Jerusalem, Israel); anti-β-actin (A2228, 1:5000) from Sigma-Aldrich (USA); and anti-myc-tag (No. 66004–1-Ig, 1:1000) and anti-HA-tag (No. 66006–1-Ig, 1:1000) from Proteintech (IL, USA). Horseradish peroxidase (HRP) conjugated anti-mouse IgG (cs7076, 1:2000) and anti-rabbit IgG (cs7074, 1:2000) secondary antibodies from Cell Signaling Technology; anti-mouse AlexaFluor488 (ab150113, 1:1000) and anti-rabbit AlexaFluor647 (ab150115, 1:1000) secondary antibodies from Abcam. N-ethylmaleimide (NEM) from Sigma-Aldrich (USA).

At 48 h after transfection, HEK293 cells were washed in phosphate buffered saline. Cells and tissue samples (stored at − 80 °C) were lysed for 30 min on ice in RIPA lysis buffer (Beyotime, Shanghai, China) plus 0.1 mmol/L phenylmethylsulfonyl fluoride and a protease inhibitor cocktail (Roche, Basel, Switzerland). Equal quantity of proteins derived from cell or tissue lysates were separated by 10% sodium dodecyl sulfate (SDS)-polyacrylamide gel electrophoresis and electro-transferred to polyvinylidene fluoride membranes, which were blocked in 5% bovine serum albumin for 1 h at room temperature, sequentially incubated with primary antibodies overnight, and the next day incubated with HRP-conjugated secondary antibodies (1:3000) for 1 h. Blots were subjected to enhanced chemiluminescence (Nikon, Japan), with β-actin or GAPDH as internal controls for total cell lysates, and Na^+^/K^+^ ATPase as an internal control for membrane proteins.

Cytoplasmic and membrane fractions were prepared using the Mem-PER™ Plus Membrane Protein Extraction Kit (89,842, Thermo Fisher Scientific, Waltham, MA, USA) according to manufacturers’ instructions and as previously described [24, 25]. Briefly, freshly prepared cells or tissue samples were washed in cell washing solution and centrifuged at 300 × *g* for 5 min. Then, permeabilization buffer was added to the pellet, vortexed briefly, and incubated for 30 min at 4 °C with constant mixing. Permeabilized cells were centrifuged for 15 min at 16,000 × *g* and supernatants containing cytoplasmic proteins were removed and transferred to new tubes. Then, pellets were suspended in solubilization buffer, incubated at 4 °C for 30 min with constant mixing, and centrifuged at 16,000 × *g* for 15 min. Supernatants containing solubilized membrane and membrane-associated proteins were transferred to new tubes. Membrane and cytoplasm fractions could be used immediately or stored at –80 °C until required.

For Co-IP, 50 μg cell or tissue lysate samples were immunoprecipitated with 1 µg anti-Cav-3 or anti-Na_v_1.5 overnight, and then incubated with 15 μl protein A/G-agarose (SC-2003, Santa Cruz, CA, USA) for 4 h at 4 °C. Agarose beads were sedimented and washed five times in cell lysis buffer, and bound proteins were released in 30 µl 2 × SDS-loading buffer. Immunoprecipitated proteins were analyzed by Western blotting.

To assess SUMOylation, 20 mmol/L NEM was added to lysis buffer for protein extraction and samples were not boiled. After Western blotting, individual bands were quantified by densitometry using ImageJ software (version 5, NIH).

### Electrocardiography (ECG)

ECG was continuously performed during studies. Surface ECG parameters were analyzed under stable baseline conditions for at least 5 min after anesthesia induction and before jugular vein preparation. P duration, PR interval, QRS duration, and corrected QT interval (QTc) were measured on standard limb lead II. ECG recordings were evaluated with LabChart software (version 7.3, AD Instruments Pty Ltd., Australia). Ventricular arrhythmias duration, episodes of ventricular tachycardia (VT), sustained VT, and ventricular fibrillation (VF) within consecutive 5 min blocks after ligation, were determined. VT comprised three or more consecutive ventricular premature beats; sustained VT was an episode lasting 10 s or more. We used the Curist–Walker scoring system to grade arrhythmia severity in rats under experimental myocardial I/R conditions [26].

### Statistical analysis

Results were presented as the mean ± standard error of the mean and analyzed by Student’s *t*-test and one-way analysis of variance (ANOVA) followed by Newman–Keuls post hoc tests. For non-normally distributed parameters, such as ventricular arrhythmia episodes and sustained VT and VF durations, Kruskal–Wallis tests were used. For ECG wave changes, two-way ANOVA was used. Percent survival rates were analyzed using the Kaplan–Meier method. GraphPad Prism 6.0 software (version 5 for Windows, San Diego, CA, USA) was used for statistical analyses. Statistical significance was accepted at *P* < 0.05.

## Results

### Experimental AAV9-mediated *PIASy* silencing model in the rat heart

**Fig. 1:**
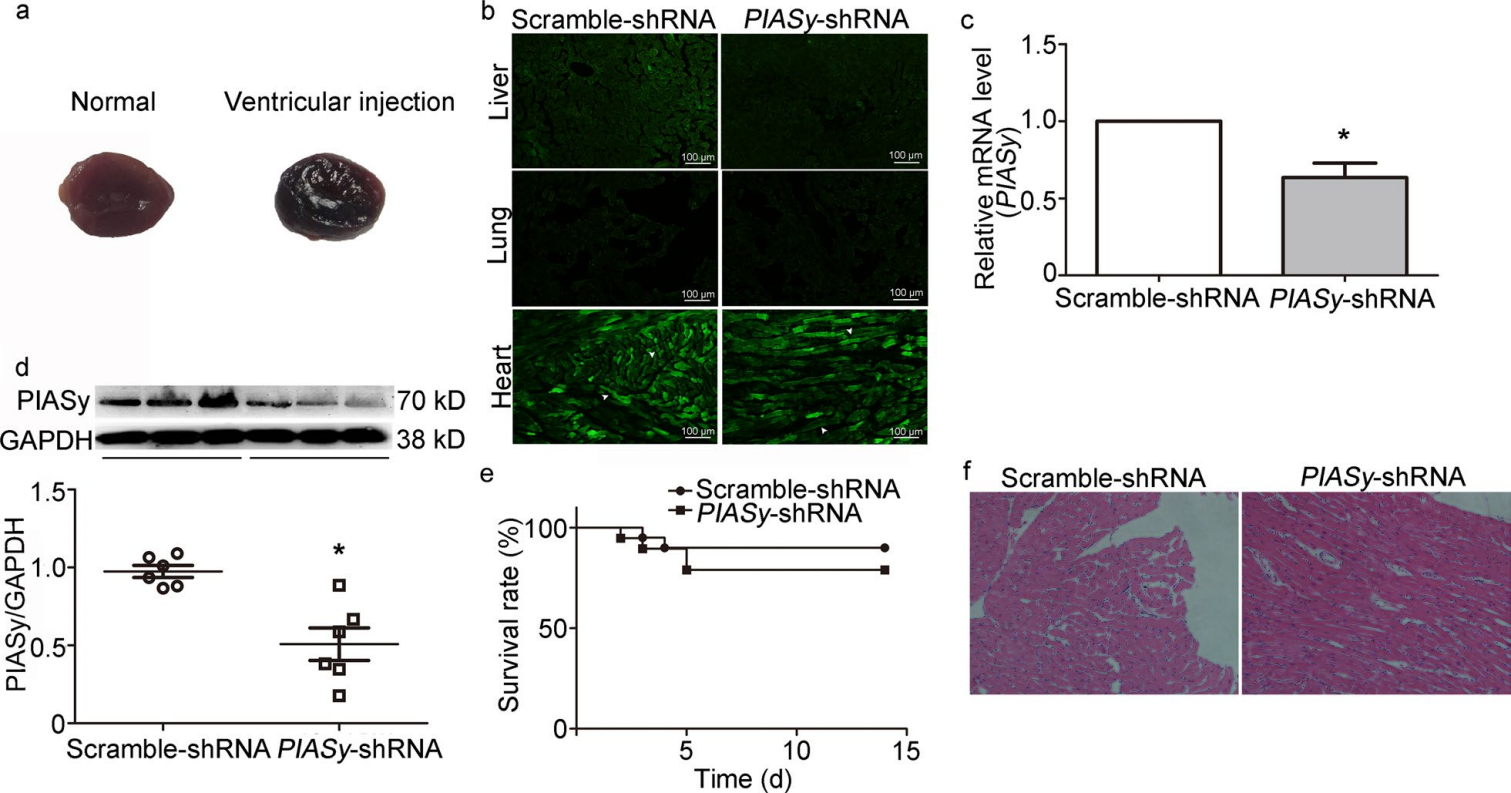
Efficiency and safety of AAV9-mediated *PIASy* silencing in the rat heart. **a** Distribution of Evans blue dye (2% v/w in 50 μl) in the rat heart at 10 min after intraventricular injection. **b** Green fluorescent protein (ZsGreen) expression mediated by the AAV9 vector in liver, lung, and heart samples at 15 d after intraventricular injection of AAV9-ZsGreen-shRNA (50 μl, 1.3 × 10^11^ vg). Arrows indicate ZsGreen expressed in the heart. **c–d** AAV9 *PIASy* shRNA-mediated *PIASy* silencing in the rat heart. RNA and protein samples from the left ventricle were generated for qRT-PCR and Western blotting, respectively, at 15 d after AAV9-ZsGreen-shRNA administration (*n* = 5). GAPDH was used as an internal control. Values are the mean ± standard error of the mean, ^*^P < 0.05 vs. Scramble-shRNA and determined by Student’s *t-*test. **e** The effect of AAV9 *PIASy* shRNA on rat survival over the 15 d following AAV9 vector treatment (*n* = 20, *P* > 0.05, Kaplan–Meier method). **f** Histological analysis after H&E staining of rat tissue samples from the left ventricle free wall at 15 d after intraventricular AAV9-ZsGreen-shRNA injection. AAV9 adeno-associated virus subtype 9, H&E hematoxylin and eosin, PIASy protein inhibitor of activated STAT Y, qRT-PCR quantitative real-time polymerase chain reaction, shRNA short hairpin RNA

To verify the efficiency of the intraventricular injection, Evans blue dye was firstly injected into left ventricle. The results showed that the Evens blue dye was evenly distributed to the whole ventricular myocardium (right panel Fig. 1a) when compared with the normal heart (left panel Fig. 1a). To achieve direct AAV9-mediated *PIASy* shRNA transfer into the adult rat heart, scramble or *PIASy* shRNAs were packed into AAV9 vectors. AAV9 capsids were directly injected into the left ventricular cavity. Two weeks after vector transfer, 6 μm short-axis apical and anterior left ventricle sections, and lung and liver tissue sections were assessed for ZsGreen expression under fluorescence microscopy. As shown in Fig. 1b, ZsGreen was predominantly expressed in the heart, especially in the mid-ventricular region, in both Scramble-shRNA and *PIASy*-shRNA groups, which confirmed vector transduction efficiency in the heart. In contrast, very low ZsGreen expression was observed in the liver and lung. Also, *PIASy* shRNA-mediated *PIASy* silencing significantly decreased the expression of PIASy mRNA and protein levels (*P* < 0.05) (Fig. 1c–d) when compared with the Scramble-shRNA group. These findings indicated that AAV9 generated robust AAV9-mediated *PIASy* shRNA expression in cardiomyocytes and mediated cardiac-specific knockdown of the target protein. To exclude potential toxic side effects from AAV9-capsids to animals, survival rates and histological changes were examined. A lower survival rate (16/20 rats) was observed in the *PIASy*-shRNA group when compared with the Scramble-shRNA group (18/20 rats), but the difference was not significant (*P* > 0.05) (Fig. 1e). We also examined H&E stained sections of the anterior left ventricle, no significant structural alterations, e.g., cardiomyocyte swelling and necrosis, myocardial filament disruption, interstitial inflammatory cell infiltration, and replacement fibrosis, were identified in the groups (Fig. 1f). These results suggested limited toxicity from AAV9 capsids in the myocardium, and that *PIASy* silencing exerted no significant effects on the myocardium without myocardial I/R.

### ***PIASy*** silencing with AAV9 ***PIASy*** shRNA improves I/R-induced Na_v_1.5 downregulation in membrane and cytoplasmic fractions

**Fig. 2:**
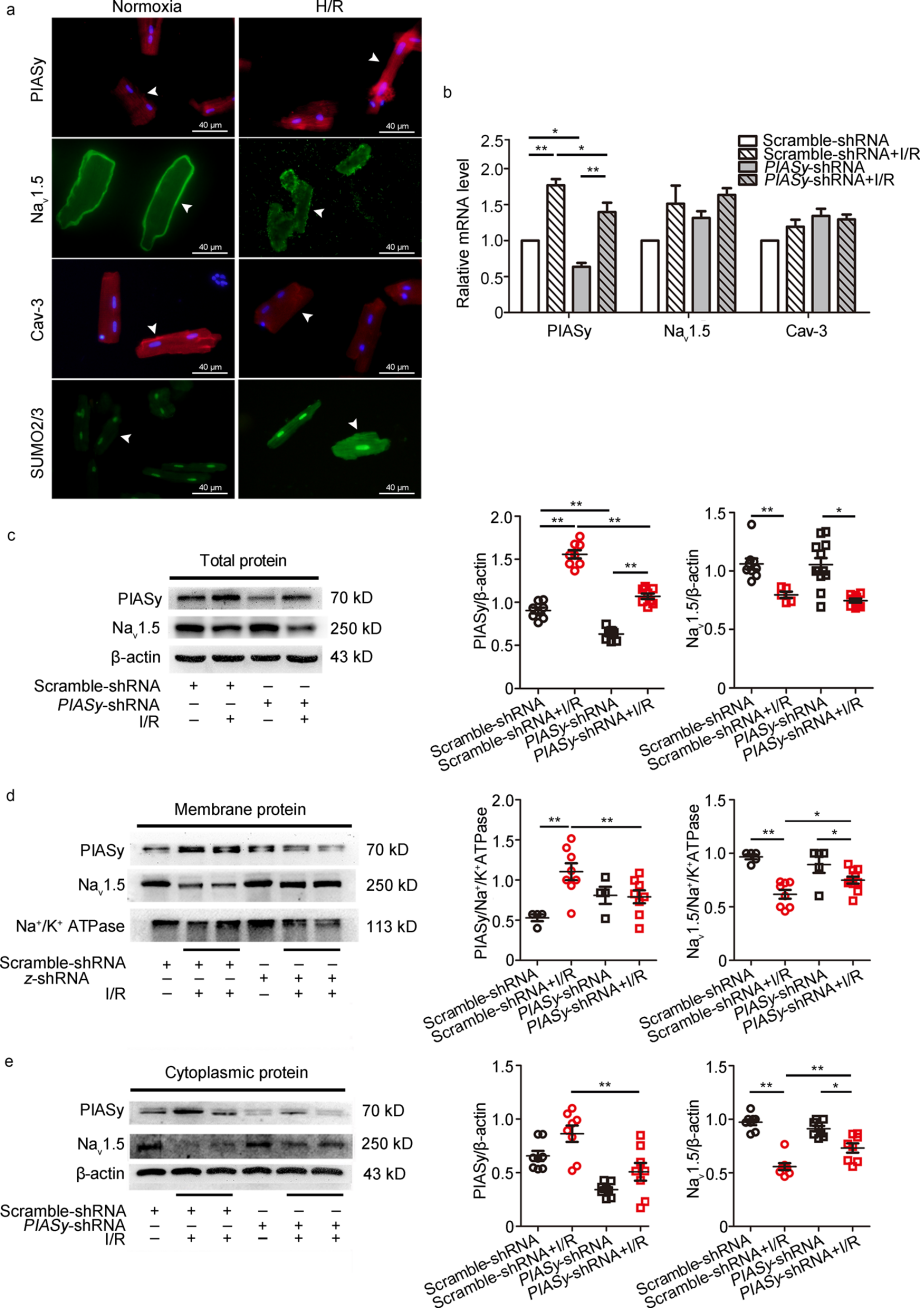
Effects of I/R and *PIASy* shRNA on PIASy, Na_v_1.5, and Cav-3 expression. **a** H/R-induced changes in PIASy, Na_v_1.5, Cav-3, and SUMO2/3 expression levels in isolated cardiomyocytes from the left ventricle of adult rats (arrows). PIASy (red) and SUMO2/3 (green) fluorescence were detected at high levels, while Na_v_1.5 (green) and Cav-3 (red) showed decreased localization, especially on membrane. **b** I/R and *PIASy* shRNA altered PIASy, Na_v_1.5 and Cav-3 mRNA levels in rat left ventricle tissues by qRT-PCR. **c–e** PIASy, Na_v_1.5, and Cav-3 protein expression in whole cardiomyocytes (**c**), membrane (**d**) and cytoplasmic (**e**) fractions after I/R in Scramble-shRNA and *PIASy*-shRNA groups. The left panel represents Western blot bands while the right represents their densitometric analysis. Values are expressed as the mean ± standard error of the mean (*n* = 10). ^*^P < 0.05, ^**^P < 0.01, one-way ANOVA. ANOVA analysis of variance, Cav-3 caveolin-3, H/R hypoxia/reoxygenation, I/R ischemia/reperfusion, Na_v_1.5 voltage-gated sodium channel 1.5, PIASy protein inhibitor of activated STAT Y, qRT-PCR quantitative real-time polymerase chain reaction, shRNA short hairpin RNA

Since PIASy was a specific E3 ligase for Cav-3, we hypothesized that it promoted SUMO conjugation to Cav-3 during I/R. As an important protein interacting with Cav-3, Na_v_1.5 was examined after I/R-induced SUMOylation of Cav-3. We first evaluated the effects of H/R on PIASy, Na_v_1.5, Cav-3, and SUMO2/3 protein levels in isolated rat cardiomyocytes. Immunofluorescence showed enhanced PIASy protein staining in cardiomyocytes exposed to 40 min hypoxia followed by 30 min reoxygenation, which was accompanied by markedly diminished Na_v_1.5 levels, predominantly in the membrane region (Fig. 2a). Furthermore, decreased Cav-3 and increased SUMO2/3 signals were also observed in H/R-treated individual cardiomyocytes when compared with normal cells (Fig. 2a). To explore the in vivo role of PIASy-mediated Cav-3 SUMOylation in Na_v_1.5 expression during I/R, we silenced *PIASy* expression in the myocardium via AAV9 *PIASy* shRNA transfection, 14 d before I/R. We extracted RNA from the myocardium in each group and analyzed PIASy, Na_v_1.5, and Cav-3 mRNA levels. No significant changes in Na_v_1.5 and Cav-3 mRNA expression levels were observed between Scramble-shRNA and *PIASy*-shRNA groups, with or without I/R (*P* > 0.05). PIASy mRNA levels were significantly higher (*P* < 0.01) after I/R in both Scramble-shRNA and *PIASy*-shRNA groups, corroborating previous findings. Although AAV9 *PIASy* shRNA decreased PIASy mRNA levels in *PIASy*-shRNA and *PIASy*-shRNA + I/R groups (*P* < 0.05, vs. Scramble-shRNA group and Scramble-shRNA + I/R group, respectively), PIASy mRNA level increases in the *PIASy*-shRNA + I/R group indicated incomplete silencing (Fig. 2b). Further lysate analyses from left ventricular peri-infarct tissue samples were performed by Western blotting. As shown in Fig. 2c–e, PIASy protein expression changes were consistent with mRNA data, especially in whole cell and cytoplasmic samples. Also, I/R induced a significant decrease in Na_v_1.5 protein expression in Scramble-shRNA and *PIASy*-shRNA groups, in whole cell, membrane, and cytoplasmic fractions (*P* < 0.05 or *P* < 0.01). However, this decrease was partially reversed by cardiac-targeted *PIASy* shRNA interference (*P* < 0.05). Though Cav-3 was modified by PIASy-mediated SUMO conjugation, Cav-3 mRNA (Fig. 2b) and protein expression (Additional file 1: Fig. S1) showed no significant changes in this model (*P* > 0.05). Since Na_v_1.5 itself was not SUMO-modified but Cav-3-regulated, further investigations are required to identify the functional interactions between Cav-3 (particularly SUMO-modified Cav-3) and Na_v_1.5, but no alteration of Cav-3 amounts, in I/R-induced Na_v_1.5 dysfunction.

### PIASy-induced Cav-3 SUMOylation by SUMO2/3 affects interactions between Cav-3 and Na_v_1.5 in vitro and in vivo

**Fig. 3:**
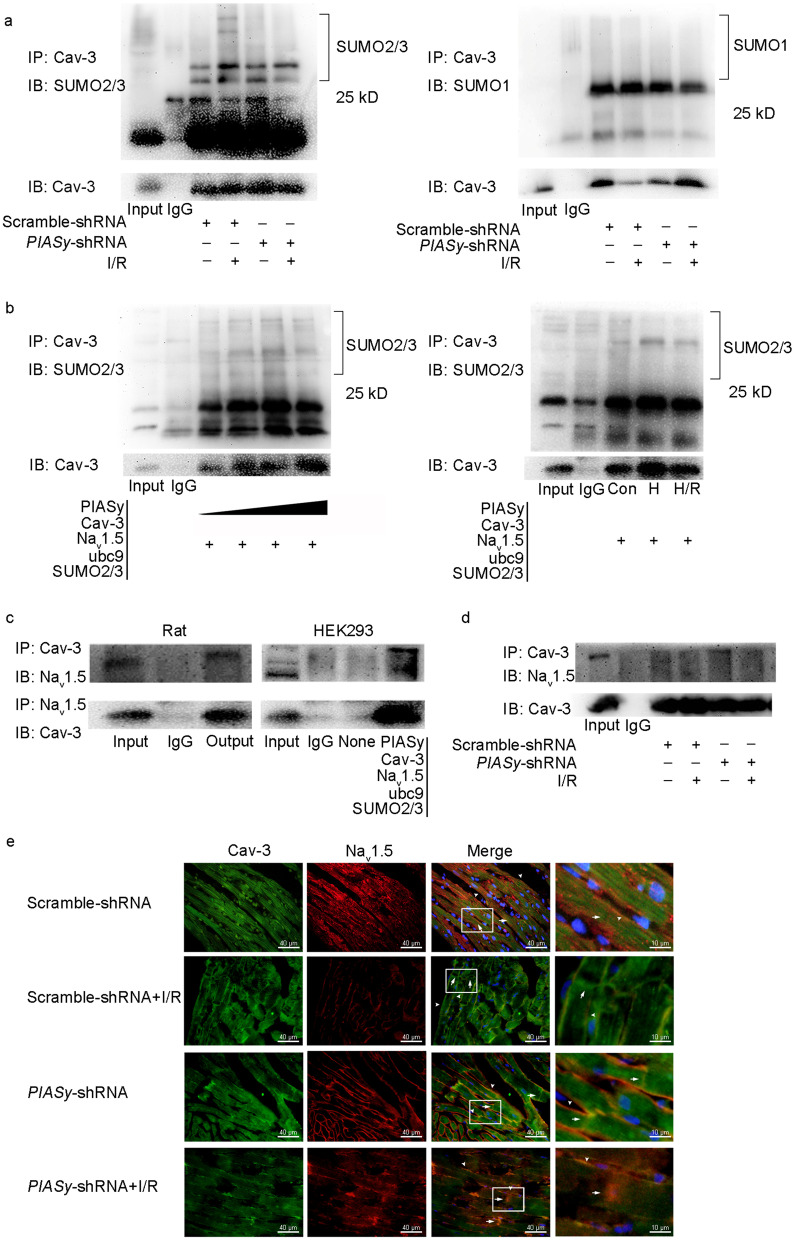
Effects of *PIASy* silencing on Cav-3 binding to SUMO2/3 and Na_v_1.5, and Na_v_1.5 distribution. **a** SUMO-modified Cav-3 in the rat heart. Rat myocardial lysates were collected and immunoprecipitated with an anti-Cav-3 antibody, then immunoblotting with anti-SUMO2/3 (left panel) or anti-SUMO1 antibodies (right panel) after I/R injury. **b** Cav-3 binding to SUMO2/3 in HEK293 cells co-transfected with Cav-3, Na_v_1.5, ubc9, SUMO2/3, and incremental doses of PIASy plasmid (left panel) or identical dose of PIASy plasmid and subjected to hypoxia or H/R (right panel). **c** Physical binding of Cav-3 and Na_v_1.5 in the rat heart, and Cav-3 and SUMO machinery in co-transfected HEK293 cells. Protein samples from normal left ventricle or HEK293 cells 48 h after transfection were immunoprecipitated with an anti-Cav-3 antibody and immunoblotted with an anti-Na_v_1.5 antibody. **d** Effects of *PIASy* shRNA on binding between Cav-3 and Na_v_1.5 by Co-IP in the I/R rat heart. **e** Cav-3 and Na_v_1.5 co-localization in rat myocardium. Immunofluorescence staining of the peri-infarct zone in the rat left ventricle showing the localization and expression levels of Na_v_1.5 (red) and Cav-3 (green), mainly on lateral membrane (arrows) and intercalated disc (arrowheads) in cardiomyocytes. Co-localization is shown in merged images. Cell nuclei were counterstained by DAPI. Scale bar = 40 μm. Cav-3 caveolin-3, Con control, Co-IP co-immunoprecipitation, DAPI 4ʹ,6-diamidino-2-phenylindole, H hypoxia, H/R hypoxia/reoxygenation, I/R ischemia/reperfusion, Na_v_1.5 voltage-gated sodium channel 1.5, PIASy protein inhibitor of activated STAT Y, shRNA short hairpin RNA, SUMO small ubiquitin-related modifier

To determine which SUMO molecule was functionally involved in Cav-3 SUMO modification in the rat myocardium, Cav-3 was precipitated from cell lysates using an anti-Cav-3 monoclonal antibody, followed by immunoblotting with antibodies specific to SUMO2/3 or SUMO1. Co-IP results showed distinct SUMO2/3-modified Cav-3 bands around 50 kD. In contrast, no visible changes in SUMO1 immunoreactive bands were identified in the Cav-3-precipitated protein. Interestingly, I/R increased SUMO2/3-modified Cav-3 levels, which were partially abrogated by *PIASy* shRNA interference (Fig. 3a). To further verify the PIASy role in modulating Cav-3 SUMOylation in vitro, HEK293 cells were transfected with plasmids expressing SUMO machinery (SUMO2/3, ubc9, and incremental PIASy doses), and also Cav-3 and Na_v_1.5 plasmids. Co-IP revealed that SUMO2/3-modified Cav-3 increased with incremental PIASy doses in precipitated Cav-3 at 48 h after transfection. Additionally, hypoxia induced a more pronounced SUMOylation of the Cav-3 protein when compared with H/R stimulation in co-transfected HEK293 cells at a stable PIASy dose (Fig. 3b). As a scaffolding protein, Cav-3 is implicated in the function and cellular translocation of several channel proteins, such as Na_v_1.5 and K_v_1.5, and may regulate their functions. To determine if molecular interactions between Cav-3 and Na_v_1.5 contributed to I/R-induced Na_v_1.5 dysfunction, reciprocal Co-IP was performed on lysates from ventricular tissue specimens and transfected HEK293 cells. As expected, decreased physical binding between Cav-3 and Na_v_1.5 was identified in vivo and in vitro (Fig. 3c). In the rat myocardium, I/R increased SUMO conjugation to Cav-3 and enhanced Cav-3 dissociation from Na_v_1.5, which was partially reversed by *PIASy* shRNA (Fig. 3d). Immunofluorescence (double staining with Cav-3 and Na_v_1.5 antibodies) in the myocardium further revealed that I/R dissociated Cav-3 with Na_v_1.5, especially in the intercalated disc and lateral membrane of cardiomyocytes (Fig. 3e). However, *PIASy* shRNA restored the co-localization of Na_v_1.5 and Cav-3 in the intercalated disc and lateral membrane (Fig. 3e). These results suggested that PIASy-mediated SUMO2/3 Cav-3 modification caused the dissociation between Cav-3 and Na_v_1.5, and consequently affected Na_v_1.5 localization and abundance during I/R.

### Mutation of SUMO consensus lysine sites in Cav-3 (K38R or K144R) alters the membrane expressions of Na_v_1.5 and Cav-3 under normal conditions and after H/R

**Fig. 4:**
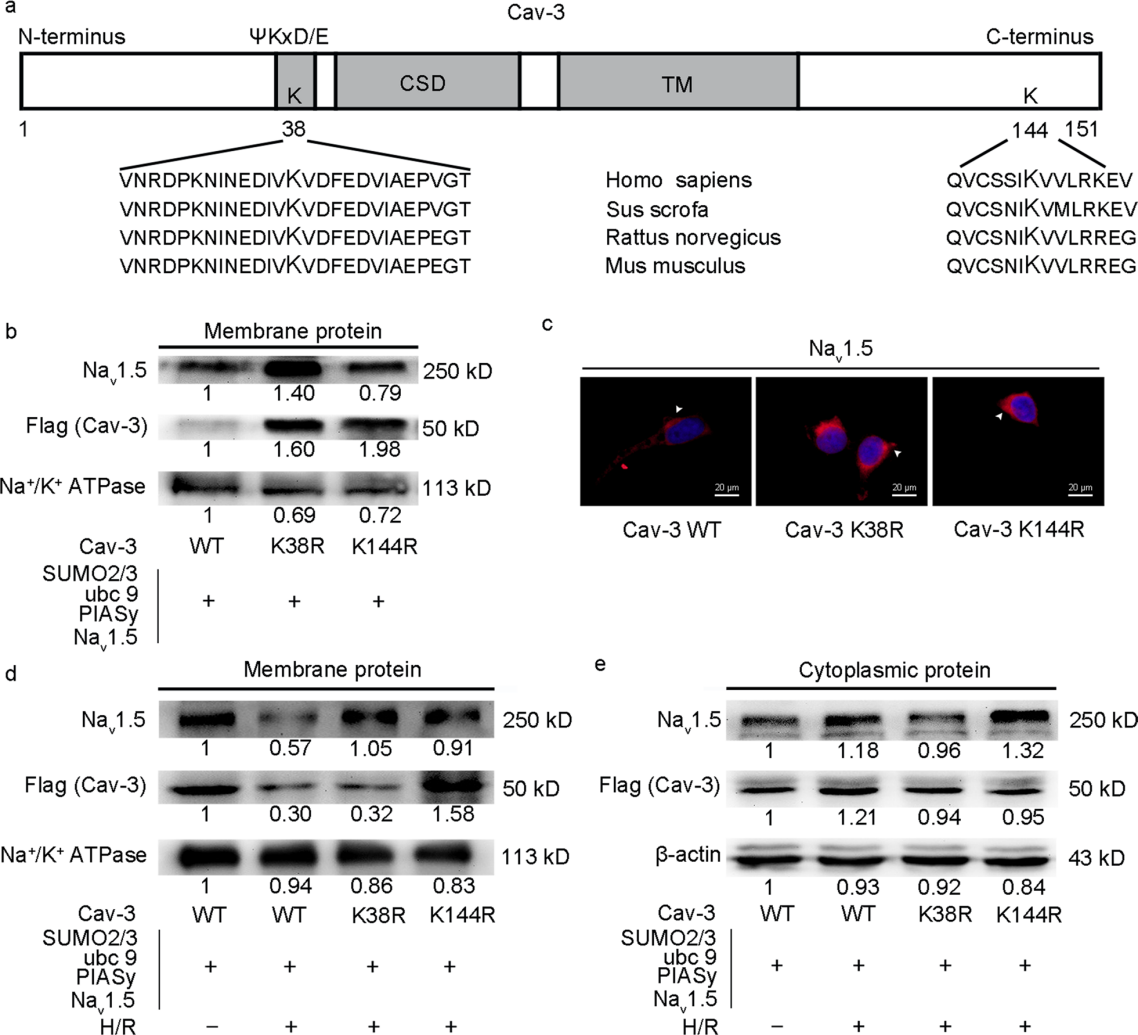
The effects of Cav-3 mutations at SUMO consensus sites on Na_v_1.5 abundance in transfected HEK293 cells. **a** Schematic showing Cav-3 SUMO consensus sites. Cav-3 has a SUMO consensus motif (ΨKxD/E) near the caveolin scaffolding domain at the N-terminus. The SUMO consensus sites K38 and K144 at the C-terminus are highly conserved across different species. **b-c** Changes in Na_v_1.5 abundance in transfected HEK293 cells between the WT and mutants. HEK293 cells were co-transfected with SUMO machinery, including HA-SUMO2/3, ubc9, Flag-hPIASy, Na_v_1.5, and Flag-tagged Cav-3 (WT, K38R mutant or K144R mutant). Membrane protein was assessed by Western blotting at 48 h after transfection. Immunofluorescence staining was performed with an anti-Na_v_1.5 antibody (red, Na_v_1.5, arrows) and DAPI (blue, nucleus). **d**–**e** Effects of Cav-3 mutations on Na_v_1.5 abundance after H/R. HEK293 cells transfected with SUMO machinery, Na_v_1.5, and Cav-3 (WT, K38R mutant or K144R mutant) were exposed to 3 h hypoxia followed by 2 h reoxygenation. Membrane and cytoplasmic proteins were immunoblotted using anti-Na_v_1.5 and anti-flag (Cav-3) antibodies, respectively. Na^+^/K^+^ ATPase and β-actin were used as internal controls for membrane and cytoplasmic proteins, respectively. Cav-3 caveolin-3, DAPI 4',6-diamidino-2-phenylindole, HEK293T human embryonic kidney 293 T cells, H/R hypoxia/reoxygenation, Na^+^/K^+^ ATPase anti-sodium potassium ATPase, Na_v_1.5 voltage-gated sodium channel 1.5, SUMO small ubiquitin-related modifier, Ubc9 ubiquitin-conjugating enzyme 9, WT wild-type

Human Cav-3 sequence analysis identified several sequences with predicted SUMOylation sites with the canonical ΨK_X_D/E sequence. One is centered on lysine-38 which lays between the N-terminus and the caveolin scaffolding domain, while the other is centered on lysine-144 located in the C-terminal tail beyond the sixth transmembrane fragment (Fig. 4a). Sequence comparisons across several vertebrate species showed that both sites are evolutionally conserved (Fig. 4a). To confirm the functional role of SUMOylated Cav-3 in Cav-3/Na_v_1.5 interactions, we mutated both lysine residues to arginine (KR mutation) and assessed membrane and cytoplasmic Na_v_1.5 levels in SUMOylation-deficient Cav-3 mutants. When the K38R plasmid was co-transfected with SUMO2/3, ubc9, PIASy, and Na_v_1.5 into HEK293 cells, membrane Na_v_1.5 protein were significantly increased when compared with the WT Cav-3 group under normal conditions. However, only slightly increased membrane Na_v_1.5 were identified for the K144R mutant (Fig. 4b). Consistent with Western blotting, immunofluorescence showed stronger Na_v_1.5 (red) signals in K38R and K144R mutant cells when compared with WT counterparts (Fig. 4c). We next examined if these SUMO mutations affected H/R-induced aberrant Na_v_1.5/Cav-3 interactions and Na_v_1.5 translocation by exposing K38R- and K144R-transfected HEK293T cells to H/R. While this significantly decreased membrane Na_v_1.5 and Cav-3 levels in WT Cav-3 cells, K38R mutation only prevented Na_v_1.5 loss in the membrane after H/R, with no visible effects on Cav-3 expression. Interestingly, the K144R mutation restored both membrane Na_v_1.5 and Cav-3 levels after H/R (Fig. 4d). Alterations in cytoplasmic Na_v_1.5 and Cav-3 were mild during H/R, and a modest increase of Na_v_1.5 in K144R-transfected cells after H/R was generated, indicating a functional role for SUMO-modified Cav-3 interactions with Na_v_1.5 during H/R (Fig. 4e).

### ***PIASy*** shRNA prevents increased phosphorylation of Na_v_1.5 fragments by I/R insult

**Fig. 5:**
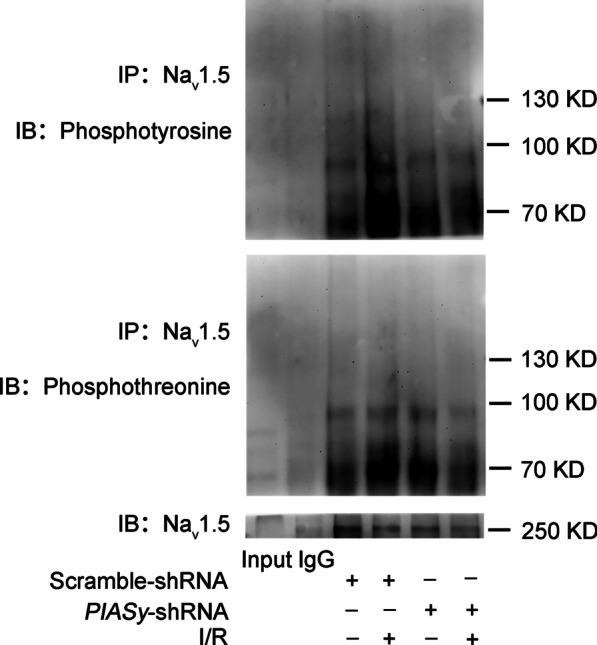
Effects of *PIASy* silencing on Na_v_1.5 phosphorylation in the I/R rat model. Rat cardiomyocyte lysates were immunoprecipitated with an anti-Na_v_1.5 antibody, followed by immunoblotting with anti-phospho-tyrosine and anti-phospho-threonine antibodies. I/R ischemia/reperfusion, Na_v_1.5 voltage-gated sodium channel 1.5, PIASy protein inhibitor of activated STAT Y, shRNA short hairpin RNA

To determine if Na_v_1.5 was phosphorylated by I/R and *PIASy* silencing, Co-IP was performed by using precipitated Na_v_1.5 from rat hearts. As shown in Fig. 5, while no phosphorylated protein bands were detected around Na_v_1.5 (about 250 kD), a significant phosphorylated band around 70–90 kD was identified, presumably from Na_v_1.5 fragments. I/R induced enhanced both phosphotyrosine and phosphothreonine bands around 70–90 kD in Na_v_1.5-precipitated heart lysates. *PIASy* silencing in rat hearts prevented increased phosphorylation of Na_v_1.5 fragments by I/R insults (Fig. 5).

### *PIASy* shRNA improves I/R-induced prolonged QTc and QRS in rats

**Fig. 6:**
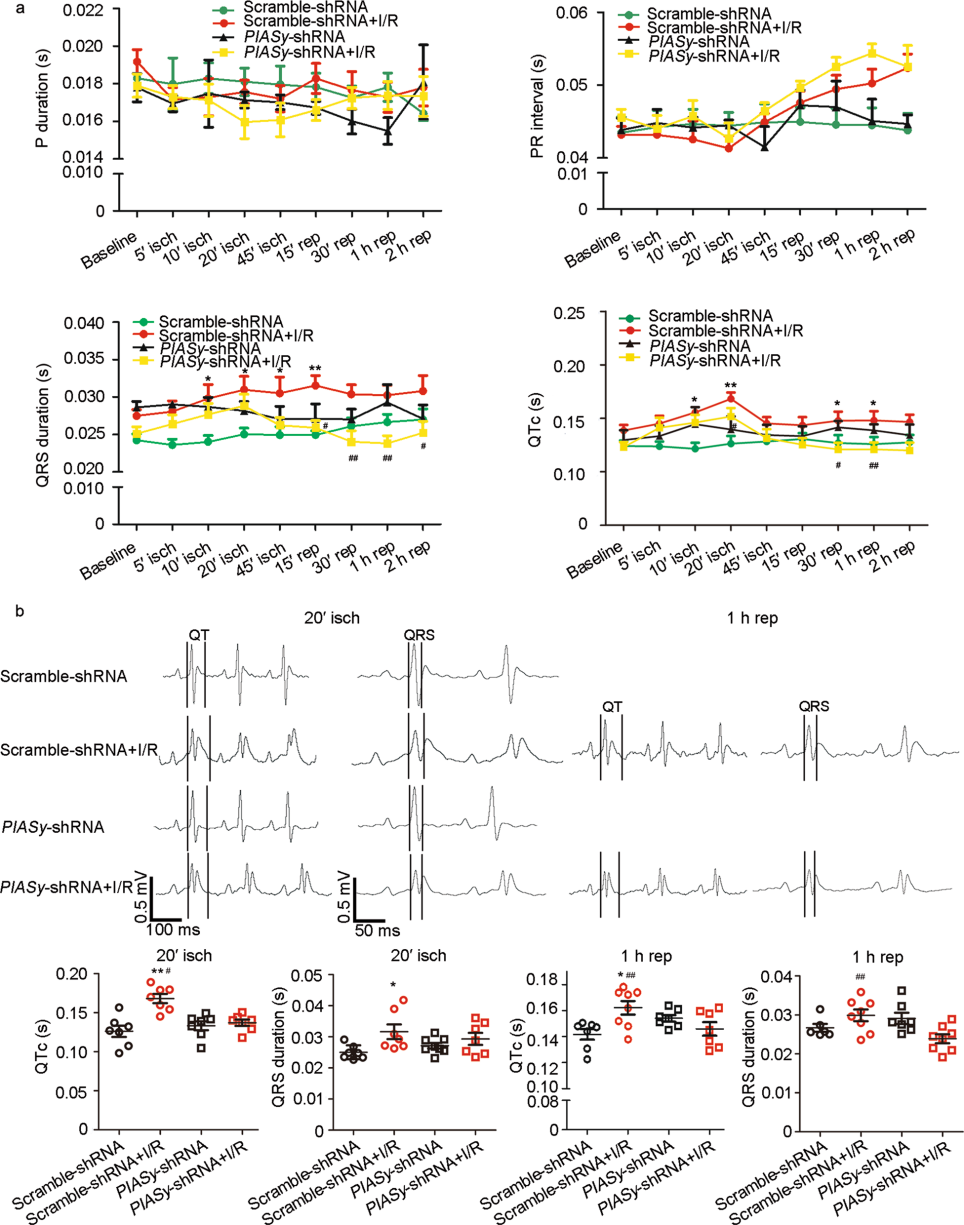
Effects of *PIASy* silencing on P duration, PR interval, QRS duration, and QTc values during I/R. **a** Changes in P duration, PR interval, QRS duration, and QTc values during I/R. **b** Representative changes in QRS waves and QT intervals (upper panel) in ECG, and statistical analyses of QRS durations and QTc values (bottom panel) at 20 min after ischemia and 1 h after reperfusion. Values are expressed as the mean ± standard error of the mean (*n* = 10). ^*^P < 0.05, ^**^P < 0.01, two-way ANOVA. ANOVA analysis of variance, ECG electrocardiography, isch ischemia, I/R ischemia/reperfusion, QTc corrected QT interval, PIASy protein inhibitor of activated STAT Y, rep reperfusion, shRNA short hairpin RNA

I/R-induced Na_v_1.5 dysregulation contributed to abnormal cardiac electrical conduction and subsequent fetal arrhythmias. ECG was performed in scramble shRNA- and *PIASy* shRNA-treated rats, with or without I/R. *PIASy* silencing caused no detectable alterations in P duration, PR interval, QRS duration, and QTc values when compared with the Scramble-shRNA group (*P* > 0.05) (Fig. 6a). Furthermore, *PIASy* shRNA shortened the I/R-induced prolongation of QTc and QRS duration (*P* < 0.05 vs. Scramble-shRNA + I/R group) (Fig. 6a, b).

### *PIASy* shRNA interference reduces lethal ventricular arrhythmias in I/R-injured rats

**Fig. 7:**
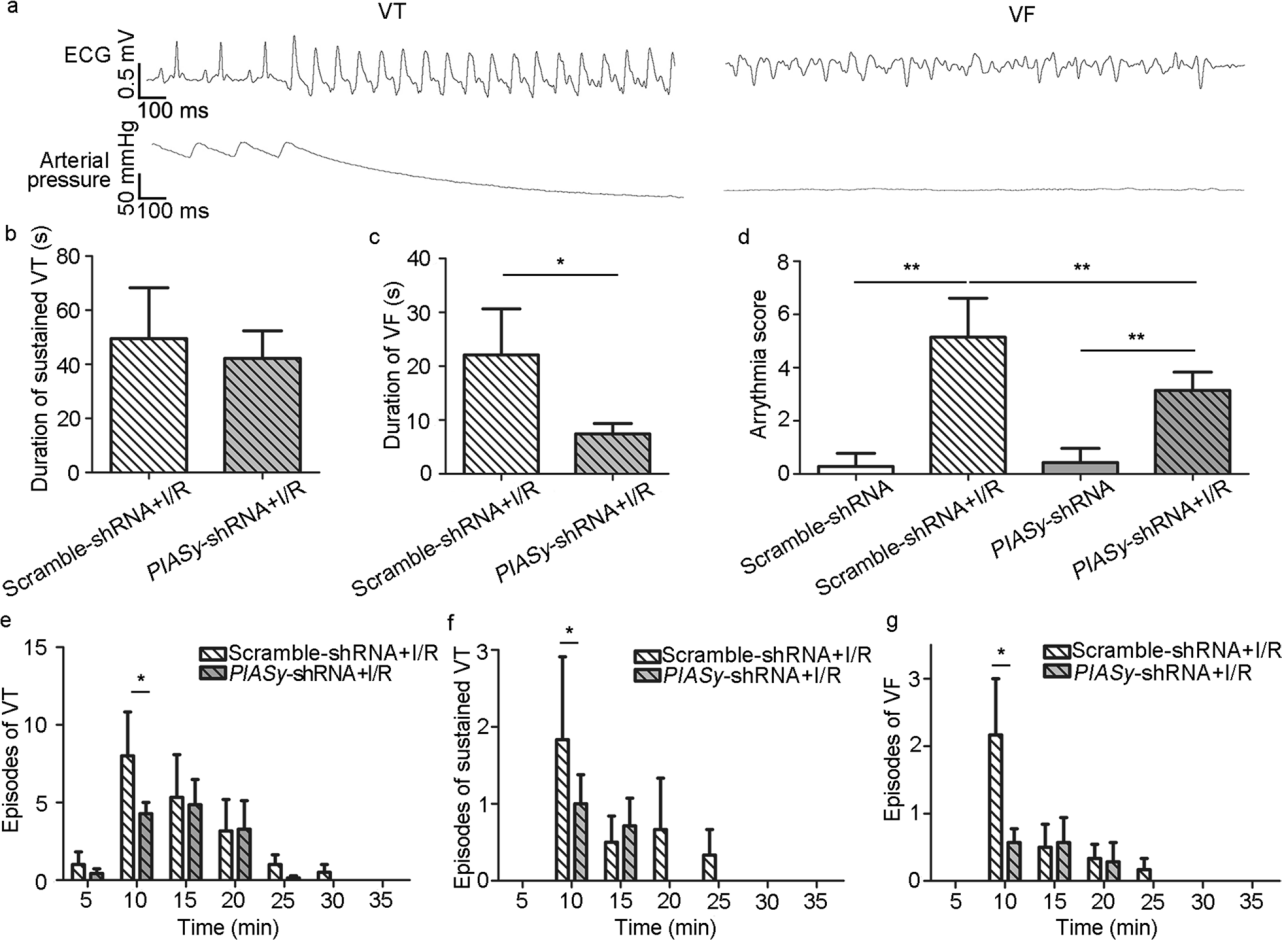
Effects of *PIASy* silencing on lethal arrythmias during myocardial I/R. **a** Schematic diagram showing VT and VF during I/R. **b-c** Duration values of sustained VT (b) and VF (c) after I/R. **d** Arrhythmia scores in I/R-injured rats with *PIASy*-shRNA or Scramble-shRNA treatments. **e–g** Episodes of VT (**e**), sustained VT (**f**), and VF (**g**) within consecutive 5 min blocks after ligation of the left anterior descending coronary artery. Values are expressed as the mean ± standard error of the mean (*n* = 10). ^*^P < 0.05, ^**^P < 0.01. Kruskal–Wallis tests were performed in **b**, **c**, and **e–g**; one-way ANOVA was performed in **d**. AAV9 adeno-associated virus subtype 9, ANOVA analysis of variance, ECG electrocardiography, I/R ischemia/reperfusion, PIASy protein inhibitor of activated STAT Y, shRNA short hairpin RNA, VF ventricular fibrillation, VT ventricular tachycardia

As shown in Fig. 7a, the episodes of VT and VF were accompanied by an obvious decrease in arterial pressure. *PIASy* shRNA transfection into rat hearts reduced I/R-induced fatal arrhythmias, which was reflected by a modest decrease in VF duration (*P* < 0.05, vs. Scramble-shRNA + I/R group), and a significantly declined arrhythmia score (*P* < 0.01, vs. Scramble-shRNA + I/R group) (Fig. 7b–d). The anti-arrhythmic effects of *PIASy* silencing were also evidenced by decreased episodes of VT and sustained VT and VF, especially at 5–10 min after ischemia (*P* < 0.05, vs. Scramble-shRNA + I/R group) (Fig. 7e–g).

## Discussion

We identified several novel findings. Firstly, using in vivo and in vitro myocardial I/R models, we demonstrated that PIASy-mediated Cav-3 SUMOylation by SUMO2/3 controlled the magnitude of Cav-3/Na_v_1.5 interactions, which affected Na_v_1.5 abundance on cardiomyocyte membranes, subsequently modulating functional cardiac conduction and lethal ventricular arrhythmias. Secondly, cardiac-targeted *PIASy* silencing mediated Cav-3 deSUMOylation and prevented I/R-induced Na_v_1.5 downregulation and ventricular arrhythmias. Additionally, using HEK293 cells transfected with SUMO machinery, mutation of SUMO consensus lysine sites in Cav-3 (K38R or K144R) altered membrane Na_v_1.5 and Cav-3 expression before and after H/R. To the best of our knowledge, this is the first study to examine such links between PIASy-related Cav-3 SUMOylation and Na_v_1.5 translocation and stability in I/R-induced fetal ventricular arrhythmias.

We showed that PIASy expression was increased both by I/R in rat hearts and by H/R in isolated rat cardiomyocytes, and was associated with reduced Na_v_1.5 membrane density. Elevated PIASy was evidenced not only on cardiomyocyte membranes but also in the cytoplasm. PIASy was previously shown to localize mainly near the nucleus, but could also be cytoplasmic [27]. It is accepted that as a SUMO E3 ligase, PIASy mediates the SUMOylation of different proteins, further regulating the activity, translocation, and autophagy of conjugated proteins [27]. As a major cardiac protein specifically localized to caveolae, Cav-3 putatively interacts with many cardiac ion channels, including Na_v_1.5 and K_v_1.5 [12]. Since no SUMO positions were identified in Na_v_1.5, which were not SUMOylated, we hypothesized that Cav-3 SUMO modifications altered its interactions with Na_v_1.5. As expected, our Co-IP data revealed that Cav-3 was physically conjugated with SUMO2/3, but not with SUMO1 in myocardial cells from the rat left ventricle. Furthermore, in vitro SUMO assays in HEK293 cells co-transfected with Cav-3 and SUMOylation machinery confirmed the presence of SUMO2/3-modified Cav-3. Interestingly, I/R, H/R, and incremental PIASy doses correspondingly increased SUMO2/3-modified Cav-3 levels. Thus, Cav-3 is the target of covalent SUMO conjugation mediated by PIASy. We also observed substantial binding between Cav-3 and Na_v_1.5 both in vivo and in vitro, with I/R possibly lessening the binding between Cav-3 and Na_v_1.5. Therefore, our data indicated that upregulated PIASy was associated with enhanced Cav-3 SUMOylation by SUMO2/3 and decreased interactions between Na_v_1.5 and Cav-3 upon I/R.

To further assess the role of PIASy-mediated Cav-3 SUMOylation in Cav-3/Na_v_1.5 interactions and plasma membrane Na_v_1.5 levels, AAV9-mediated shRNA cardiac transfections were used to interfere PIASy expression. AAV9 is a safe and useful gene therapy vector and mediates efficient cardiac-targeted interference even by intravenous delivery [28, 29]. Previous study reported that AAV9-shRNA achieved highly efficient and stable cardiac-selective knockdown in mouse and rat hearts [23]. In previous research, targeted gene expression decreased at about 7 d after transfection by AAV9-shRNA silencing, and reached the peak effect at approximately 14 d [23]. Consistent with previous findings [29], our intraventricular AAV9 vector injections markedly reduced target gene expression in adult rat hearts as evidenced by reduced PIASy mRNA and protein levels in left ventricular tissue. In AAV9 *PIASy* shRNA animals, PIASy protein expression was significantly reduced under normal and I/R conditions, and was accompanied by decreased SUMO2/3-modified Cav-3 and Cav-3/Na_v_1.5 dissociation, with increased Na_v_1.5 after I/R. In addition to in vivo PIASy knockdown by AAV9 shRNA interference to reduce SUMOylation, SUMOylation-deficient Cav-3 mutants (K38R and K144R) were examined in HEK293 cells heterologously transfected with Na_v_1.5 and SUMOylation machinery. Cav-3 has conserved SUMO consensus sites, suggesting potential targets for SUMOylation. Of these, Lys-38 was the preferred SUMOylation site for poly-SUMO3 chains, with PIASy required for its SUMOylation. Lys-38 and Lys-144 mutations, respectively, partially reversed H/R-induced decreases in Na_v_1.5, especially on cell membrane, while increasing Na_v_1.5 protein levels in membrane when compared with WT Cav-3 under normal conditions. These findings suggested that mutating SUMO sites in Cav-3 affected Na_v_1.5/Cav-3 interactions and Na_v_1.5 translocation, increased Cav-3 SUMOylation by PIASy upon I/R, and contributed to Cav-3 dissociation from Na_v_1.5, resulting in altered Na_v_1.5 density in plasma membrane.

As an important caveolin, Cav-3 played pivotal role in cardioprotection by interacting with several ion channels and signaling molecules via its scaffold domains [30]. Our previous study reported that Cav-3 was essential for propofol-induced cardiac protection against I/R injury [31]. Accumulating evidence also indicated that Cav-3 putatively regulated Na_v_1.5 activity by binding to Na_v_1.5 [32]. The physical binding of Cav-3 and Na_v_1.5 was also supported by our Co-IP data in rat heart tissue samples and heterologously transfected cells. The function of Cav-3 in Na_v_1.5 activity was further confirmed by evidence showing that mutations in specific Cav-3 sites were associated with dysregulated Na_v_1.5 activity and severe arrhythmias, such as long QT syndrome [33]. However, how Cav-3 modifications affected its interactions with Na_v_1.5 remains elusive. Since protein SUMOylation emerged as an important protein modification strategy for functional regulation in diverse cellular processes, including protein localization, stability, and stress responses, more E3 SUMO ligases such as the PIAS protein family, NSE2, and EGR2 have been identified [34]. As stated, PIASy was important in terms of its ability to regulate Cav-3 SUMOylation in response to I/R stimulation. Similar roles for PIASy were reported in regulating specific molecules, including von Hippel-Lindan [35], NF-kappaB essential modulator [36], p53 [37], and Tat-interacting protein 60 [38] in response to different cellular environments.

Although SUMOylated Cav-3 altered Na_v_1.5 levels in cardiomyocytes, the underlying mechanisms remains unclear. Decreased Na_v_1.5 expression induced by I/R was evidenced not only in the plasma membrane fraction, but also in the cytoplasmic fraction. These results suggested the involvement of Na_v_1.5 localization and degradation. Recent findings showed that Na_v_1.5 phosphorylation at specific sites regulated its intracellular translocation and activity [39]. Also, protein kinase A (PKA)-dependent phosphorylation at S526 or S529 sites in ID I–II promoted Na_v_1.5 trafficking to the plasma membrane [40], while protein kinase C (PKC)-dependent phosphorylation at S1503 decreased its membrane levels [41]. We observed increased Na_v_1.5 phosphorylation after I/R, which was linked to reduced membrane Na_v_1.5 levels. In vivo *PIASy* silencing blunted I/R-induced Na_v_1.5 phosphorylation and restored membrane Na_v_1.5 abundance. These data indicated that activating Na_v_1.5 phosphorylation following its dissociation with SUMO-regulated Cav-3 may have roles for its trafficking away from the membrane. Na_v_1.5 is ubiquitinated by UBR3/6 and is subsequently degraded via the ubiquitin-protease system [42]. While we observed that enhanced Cav-3 SUMO was associated with decreased Na_v_1.5 binding to Cav-3 and Na_v_1.5 downregulation, no active ubiquitination of Na_v_1.5 was detected in I/R-treated rat hearts (data not shown). Whether other post-transcriptional events, such as dysfunctional trafficking and endocytosis, contribute to decreased membrane Na_v_1.5 expression during I/R remains unclear. The precise mechanisms whereby PIASy promotes Na_v_1.5 dissociation from Cav-3 and facilitates Na_v_1.5 translocation during I/R require further investigation.

As the main sodium channel subunit for cardiomyocyte action potential development, Na_v_1.5 plays pivotal roles in cardiac conduction. Abnormal Na_v_1.5 expression and distribution may exert deficient Na^+^ currents and conduction, which are arrhythmogenic [43]. Reduced Na_v_1.5 protein expression after I/R, primarily on cardiomyocyte membrane, prolonged the QRS complex due to deficient conduction and lethal ventricular arrhythmias as previously described [21]. In this study, we showed that *PIASy* silencing prevented I/R-induced loss of membrane Na_v_1.5, QRS duration increase, elevated arrhythmia scores, VF duration, and episodes of sustained VT and VF. It is noteworthy that enhanced late sodium current (*I*_NaL_) induced by I/R is another mechanism underlying fatal arrhythmias; *I*_NaL_ is generated by delayed inactivation or reopen of cardiac sodium channels and prolongs QTc [44]. The inhibition of *I*_NaL_ by ranolazine reversed I/R-induced QTc prolongation and effectively ameliorated ventricular arrhythmias [45]. In our study, *PIASy* shRNA silencing showed similar effects as ranolazine, suggesting that PIASy may affect both *I*_NaL_ and Na_v_1.5 expression. Further investigations are required to determine how PIASy regulates *I*_NaL_ and Na_v_1.5 functions in pathological circumstances such as I/R and heart failure.

Our study had some limitations. Firstly, given that arrhythmias mainly occur in the ischemic phase, and it takes time after AAV9 injection for peak protein silencing, we injected vectors 14 d before I/R. If vectors are injected after I/R, the effects of *PIASy* silencing on chronic myocardial injury induced by I/R should be investigated in future studies. Secondly, since no studies have reported how AAV9 affects PIASy expression, it is important to involve non-genetically engineered hearts as controls. Future studies are required to interpret these concerns.

## Conclusions

**Fig. 8:**
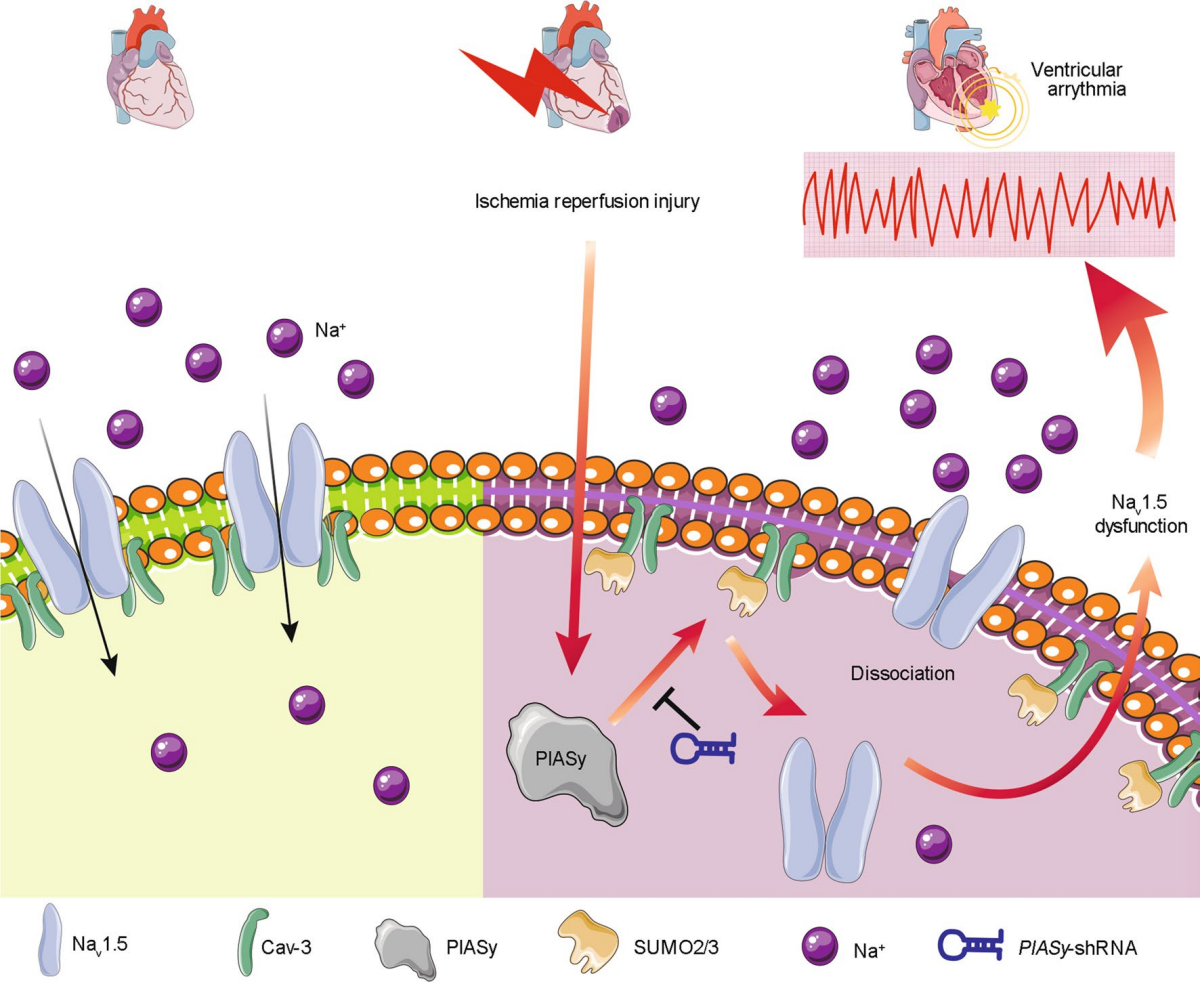
Schematic showing proposed signaling involving PIASy-mediated Cav-3 SUMOylation effects on reperfusion arrhythmias. PIASy-mediated Cav-3 SUMOylation by SUMO2/3 controls the magnitude of Cav-3/Na_v_1.5 interaction, which affects Na_v_1.5 abundance on cardiomyocyte membrane and modulate lethal reperfusion arrhythmias. Cav-3 caveolin-3, Na_v_1.5 voltage-gated sodium channel 1.5, PIASy protein inhibitor of activated STAT Y, shRNA short hairpin RNA

We identified novel links between PIASy-related Cav-3 SUMOylation and Na_v_1.5 translocation and stability, and determined a new mechanism underpinning I/R-induced Na_v_1.5 downregulation and fatal ventricular arrhythmias (Fig. 8). *PIASy* silencing by shRNA prevented Cav-3 dissociation from Na_v_1.5 and membrane Na_v_1.5 reductions after I/R. We identified PIASy as a potential therapeutic target for life-threatening arrhythmias in patients with ischemic heart disease.

## Supplementary Information

**Additional file 1: Fig. S1**. Effects of I/R and PIASy shRNA on the protein expression of Cav-3 in rat left ventricle tissues.

## Data Availability

The data and materials used to support the findings of this study are available from the corresponding authors upon request.

## Abbreviations

AAV9: Adeno-associated virus subtype 9
ANOVA: Analysis of variance
Cav-3: Caveolin-3
Co-IP: Co-immunoprecipitation
DAPI: 4′6-Diamidino-2-phenylindole
ECG: Electrocardiography
H&E: Hematoxylin and eosin
H/R: Hypoxia/reoxygenation
HEK293T: Human embryonic kidney 293 T cells
HRP: Horseradish peroxidase
I/R: Ischemia/reperfusion
LAD: Left anterior descending coronary artery
Na^+^/K^+^ ATPase: Anti-sodium potassium ATPase
Na_v_1.5: Voltage-gated sodium channel 1.5
NEM: N-Ethylmaleimide
PIASy: Protein inhibitor of activated STAT Y
qRT-PCR: Quantitative real-time polymerase chain reaction
QTc: Corrected QT interval
SDS: Sodium dodecyl sulfate
shRNA: Short hairpin RNA
SUMO: Small ubiquitin-related modifier
Ubc9: Ubiquitin-conjugating enzyme 9
VF: Ventricular fibrillation
VT: Ventricular tachycardia
WT: Wild-type
