# Supplementary material for: Cardiac-targeted PIASy gene silencing mediates deSUMOylation of caveolin-3 and prevents ischemia/reperfusion-induced Nav1.5 downregulation and ventricular arrhythmias
Source: Mil Med Res. 2022 Oct 14;9:58. doi: 10.1186/s40779-022-00415-x (PMC9563440; doi:10.1186/s40779-022-00415-x)
Supplement: Supplementary file 1 — Additional file 1: Fig. S1. Effects of I/R and PIASy shRNA on the protein expression of Cav-3 in rat left ventricle tissues. [file 40779_2022_415_MOESM1_ESM.pdf]

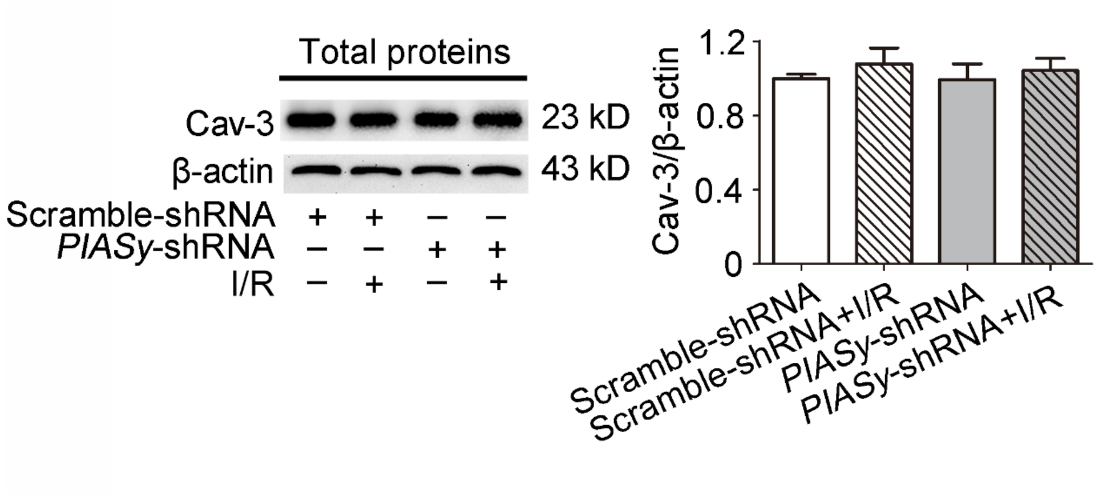

Fig. S1 Effects of I/R and *PIASy* shRNA on the protein expression of Cav-3 in rat left ventricle tissues. The left panel represents Western blotting bands while the right represents their densitometric analysis. β-actin was used as internal control. Values are expressed as the mean ± standard error of the mean (n = 10). Cav-3 caveolin-3, I/R ischemia/reperfusion, PIASy protein inhibitor of activated STAT Y, shRNA short hairpin RNA
